# Momelotinib versus Continued Ruxolitinib or Best Available Therapy in JAK Inhibitor-Experienced Patients with Myelofibrosis and Anemia: Subgroup Analysis of SIMPLIFY-2

**DOI:** 10.1007/s12325-024-02928-4

**Published:** 2024-07-11

**Authors:** Claire N. Harrison, Alessandro M. Vannucchi, Christian Recher, Francesco Passamonti, Aaron T. Gerds, Juan Carlos Hernandez-Boluda, Abdulraheem Yacoub, Shireen Sirhan, Catherine Ellis, Bharat Patel, Bryan Strouse, Uwe Platzbecker

**Affiliations:** 1 Guy’s and St Thomas’ NHS Foundation Trust, Great Maze Pond, London, SE1 9RT UK; 2 University of Florence, Florence, Italy; 3 University Hospital Center (CHU) of Toulouse, Toulouse, France; 4 Fondazione IRCCS Ca’ Granda Ospedale Maggiore Policlinico, Dipartimento di Oncologia ed Onco-Ematologia, Università degli Studi di Milano, Milan, Italy; 5 Cleveland Clinic Taussig Cancer Institute, Cleveland, OH USA; 6 Hospital Clìnico de Valencia-INCLIVA, University of Medicine, Valencia, Spain; 7 University of Kansas, Kansas City, KS USA; 8 Jewish General Hospital, McGill University, Montreal, QC Canada; 9 GSK plc, Philadelphia, PA USA; 10 University Hospital Leipzig, Leipzig, Germany

**Keywords:** Anemia, Anemia supportive therapy, Erythropoiesis-stimulating agents, Hemoglobin, Momelotinib, Myelofibrosis, Red blood cell transfusions, Ruxolitinib, Transfusion independence

## Abstract

**Introduction:**

Some Janus kinase (JAK) inhibitors such as ruxolitinib and fedratinib do not address and may worsen anemia in patients with myelofibrosis. In these cases, the JAK inhibitor may be continued at a reduced dose in an effort to maintain splenic and symptom control, with supportive therapy and/or red blood cell (RBC) transfusions added to manage anemia. This post hoc descriptive analysis of the phase 3 SIMPLIFY-2 trial evaluated the relative benefits of this approach versus switching to the JAK1/JAK2/activin A receptor type 1 inhibitor momelotinib in patients for whom anemia management is a key consideration.

**Methods:**

SIMPLIFY-2 was a randomized (2:1), open-label, phase 3 trial of momelotinib versus best available therapy (BAT; 88.5% continued ruxolitinib) in JAK inhibitor-experienced patients with myelofibrosis (*n* = 156). Patient subgroups (*n* = 105 each) were defined by either baseline (1) hemoglobin (Hb) of < 100 g/L or (2) non-transfusion independence (not meeting the criteria of no transfusions and no Hb of < 80 g/L for the previous 12 weeks); outcomes have been summarized descriptively.

**Results:**

In both subgroups of interest, week 24 transfusion independence rates were higher with momelotinib versus BAT/ruxolitinib: baseline Hb of < 100 g/L, 22 (33.3%) versus 5 (12.8%); baseline non-transfusion independent, 25 (34.7%) versus 1 (3.0%). Mean Hb levels over time were also generally higher in both subgroups with momelotinib, despite median transfusion rates through week 24 with momelotinib being comparable to or lower than with BAT/ruxolitinib. Spleen and symptom response rates with momelotinib in these subgroups were comparable to the intent-to-treat population, while rates with BAT/ruxolitinib were lower.

**Conclusion:**

In patients with moderate-to-severe anemia and/or in need of RBC transfusions, outcomes were improved by switching to momelotinib rather than continuing ruxolitinib and using anemia supportive therapies.

**Trial Registration:**

ClinicalTrials.gov: NCT02101268.

**Supplementary Information:**

The online version contains supplementary material available at 10.1007/s12325-024-02928-4.

## Plain Language Summary

Patients with the rare blood cancer myelofibrosis often experience symptoms such as tiredness, an increase in the size of their spleens (an organ involved in filtering the blood), and anemia (too few red blood cells). One type of treatment for myelofibrosis, called a Janus kinase (JAK) inhibitor, can help patients to feel better and reduce the size of their spleens, but some JAK inhibitors do not help with anemia and may make it worse. In those situations, patients may continue to take their JAK inhibitor but also receive another type of treatment, called an anemia supportive therapy, and may also receive red blood cell transfusions. This study compared 2 treatment approaches, continuing the JAK inhibitor ruxolitinib and adding an anemia supportive therapy and/or transfusions versus switching to another treatment called momelotinib, in 2 groups of patients from a clinical trial: (1) patients with levels of hemoglobin (a red blood cell protein) at the start of the trial that indicated that they had anemia, and (2) patients who were already receiving red blood cell transfusions at the start of the trial. In both groups, more patients did not need red blood cell transfusions anymore at week 24 with momelotinib, and their hemoglobin levels on average became higher over time. More patients also had improvements in spleen size and symptoms with momelotinib. Overall, outcomes were improved by switching to momelotinib rather than continuing ruxolitinib and using supportive therapies and/or red blood cell transfusions to treat anemia.

**Supplementary Information:**

The online version contains supplementary material available at 10.1007/s12325-024-02928-4.

## Key Summary Points

**Table:** 

Although Janus kinase (JAK) inhibitors are the mainstay of myelofibrosis treatment for their ability to improve spleen size and symptoms, some, such as ruxolitinib, do not address, and may worsen, anemia; supportive therapies—such as erythropoiesis-stimulating agents (ESAs), androgens, and immunomodulators—and/or red blood cell (RBC) transfusions are often added to reduced-dose ruxolitinib to mitigate anemia, but the efficacy of this approach may be suboptimal
This post hoc descriptive analysis of the phase 3 SIMPLIFY-2 study of momelotinib versus best available therapy (BAT; 88.5% ruxolitinib) in JAK inhibitor-experienced patients with myelofibrosis was conducted to evaluate whether outcomes in patient subgroups for whom anemia management is a key consideration are improved by switching to momelotinib versus the traditional approach of continuing ruxolitinib and adding anemia supportive therapies and/or RBC transfusions
Anemia-related benefits, including week 24 transfusion independence rates and mean hemoglobin levels over time, were higher with momelotinib versus BAT/ruxolitinib in both subgroups evaluated (patients with baseline hemoglobin of < 100 g/L and patients who were non-transfusion independent at baseline); spleen and symptom benefits with momelotinib in these subgroups were consistent with the intent-to-treat population but lower with BAT/ruxolitinib
In JAK inhibitor-experienced patients who require anemia management, switching to momelotinib improves anemia-related outcomes while maintaining spleen and symptom responses in contrast to the lower transfusion independence, spleen, and symptom response rates observed with continued ruxolitinib plus anemia supportive therapies and/or RBC transfusions

## Introduction

Anemia represents a substantial medical need in myelofibrosis; approximately one-third of patients have hemoglobin (Hb) of < 100 g/L at diagnosis, and nearly all become anemic over time [1, 2]. Many patients receive red blood cell (RBC) transfusions to manage anemia, but transfusion dependency is associated with diminished quality of life and is a negative prognostic factor for survival [1]. While Janus kinase (JAK) inhibitors are the backbone of myelofibrosis treatment due to their efficacy in managing symptoms and splenomegaly, some, such as ruxolitinib and fedratinib, do not alleviate, and may exacerbate, anemia [3–7]. In the absence of therapies that directly address the mechanisms of anemia in myelofibrosis—including increased pro-inflammatory signaling, RBC sequestration, and dysregulated iron metabolism [1]—anemia supportive therapies such as erythropoiesis-stimulating agents (ESAs; e.g., epoetin alfa), androgens (e.g., danazol), corticosteroids, and immunomodulatory drugs have been traditionally added in combination with continued ruxolitinib or fedratinib to manage new or worsening anemia [1, 3]. However, these adjunct treatments offer limited efficacy, durability, and tolerability, while dose reduction of the concomitant JAK inhibitor to mitigate myelosuppressive effects may compromise clinical benefit [3].

Momelotinib, a JAK1/JAK2/activin A receptor type 1 (ACVR1) inhibitor approved specifically for the treatment of patients with myelofibrosis who have anemia [7], has demonstrated clinically meaningful and durable improvements in anemia, splenomegaly, and symptoms in both JAK inhibitor-naive and -experienced patients with myelofibrosis across 3 phase 3 trials [8–10]. Thus, both JAK inhibitor-naive patients who already present with disease-related anemia and JAK inhibitor-experienced patients who develop new or worsening anemia on ruxolitinib or fedratinib may benefit from momelotinib. While clinical guidelines include momelotinib for patients with myelofibrosis and anemia, traditional anemia supportive therapies continue to be considered as well, particularly for patients who also have splenomegaly and constitutional symptoms that are well controlled on an alternative first-line JAK inhibitor [11].

In the phase 3 SIMPLIFY-2 trial, momelotinib was evaluated versus best available therapy (BAT) in JAK inhibitor-experienced patients who had experienced hematologic toxicity while receiving ruxolitinib [9]. While the primary endpoint of superiority in the rate of spleen volume reduction ≥ 35% from baseline at week 24 with momelotinib versus BAT was not met (6.7% vs. 5.8%; *P* = 0.90), momelotinib was associated with nominally significant improvements in the rates of total symptom score (TSS) reduction ≥ 50% (26.2% vs. 5.9%; nominal *P* = 0.0006) and RBC transfusion independence at week 24 (43.3% vs. 21.2%; nominal *P* = 0.0012) [9]. Notably, treatment washout from prior ruxolitinib was not permitted, and 88.5% of patients in the BAT arm remained on ruxolitinib, including some who continued to receive therapeutic doses [9]. Thus, this trial provides an opportunity to evaluate the anemia-related benefits of switching to momelotinib versus continuing ruxolitinib and managing anemia with supportive therapies and/or RBC transfusions. To that end, we performed a post hoc descriptive analysis of SIMPLIFY-2 focused on patient subgroups for whom anemia management is a key consideration: (1) patients with moderate-to-severe anemia, defined by baseline Hb of < 100 g/L, and (2) patients who were not transfusion independent (TI) at baseline.

## Methods

### Study Design

SIMPLIFY-2 (NCT02101268) was a multicenter, open-label, phase 3 study in which patients were randomized 2:1 to receive momelotinib or BAT [9]. It was conducted in accordance with the Declaration of Helsinki and Good Clinical Practice. The study protocol was approved by the institutional review board or independent ethics committee at each study site, and all participants provided written informed consent.

Consistent with the primary analysis [9], the present analyses are based on the 24-week randomized period, during which patients received either momelotinib 200 mg once daily or BAT according to the standard of care and investigator discretion. Clinical assessments, including laboratory values, were every 2 weeks, and abdominal scans for spleen size were every 12 weeks. The modified Myeloproliferative Neoplasm Symptom Assessment Form TSS electronic diary was self-completed by patients daily, and all transfusions were recorded. After the primary endpoint was assessed at week 24 [9], all patients were eligible to receive open-label momelotinib.

### Patients

A total of 156 patients were randomized to receive momelotinib (*n* = 104) or BAT (*n* = 52); full inclusion and exclusion criteria have been previously described [9]. Eligible patients were ≥ 18 years of age with primary, post-polycythemia vera, or post-essential thrombocythemia myelofibrosis classified as high, intermediate-2, or intermediate-1 (with symptomatic splenomegaly or hepatomegaly) risk (per Dynamic International Prognostic Scoring System criteria); there was no minimum platelet count requirement. All patients were currently or previously treated with ruxolitinib for ≥ 28 days and either required RBC transfusions or dose adjustment of ruxolitinib to < 20 mg twice daily with grade ≥ 3 anemia, thrombocytopenia, or bleeding on treatment; no washout of ruxolitinib was required prior to enrollment. Stratification factors included transfusion dependence (yes or no) and TSS (< 18 or ≥ 18).

The patient subgroups for the present analysis were defined post hoc by either baseline (1) Hb of < 100 g/L or (2) transfusion status, namely all patients who were non-TI. Per the study protocol, baseline transfusion independence was defined as the absence of RBC transfusions and no Hb of < 80 g/L in the 12 weeks before randomization, while transfusion dependence was defined as ≥ 4 RBC units transfused or an Hb of < 80 g/L in the 8 weeks before randomization. Thus, the baseline non-TI subgroup in the present analysis comprised patients who either met these criteria for transfusion dependence or were transfusion requiring (TR; received transfusions but did not meet the criteria for either transfusion independence or dependence).

As few patients received anemia supportive therapies other than RBC transfusions (e.g., ESAs), results in the small subgroup of the overall intent-to-treat (ITT) population who did were also summarized.

### Endpoints and Statistical Analysis

The primary endpoint (superiority) was splenic response rate (spleen volume reduction ≥ 35% from baseline) at week 24. Secondary endpoints included TSS response rate (≥ 50% reduction from baseline) and transfusion independence rate (no RBC transfusions or Hb of < 80 g/L in the last 12 weeks before week 24). TSS was assessed in patients with baseline TSS of > 0 or baseline TSS of 0 but missing or > 0 at week 24. In addition to the prespecified terminal 12-week definition of transfusion independence at week 24, transfusion independence by week 24 was also assessed post hoc using a rolling 12-week definition (no RBC transfusions or Hb of < 80 g/L during any 12-week period through week 24). All statistical power was used at the time of the primary analysis [9]; efficacy and safety results from this exploratory analysis are summarized descriptively.

## Results

### Baseline Characteristics, Dosing, and Safety

Overall baseline characteristics in these subgroups of interest, other than those related to Hb level and/or transfusion status, were consistent with the ITT population [9]. Mean duration of prior ruxolitinib was > 1 year across all arms and subgroups (Table 1).

**Table 1:** Baseline characteristics of patients in the baseline Hb < 100 g/L and non-TI subgroups

	Baseline Hb < 100 g/L		Baseline non-TI	
	Momelotinib (*n* = 66)	BAT/RUX (*n* = 39)	Momelotinib (*n* = 72)	BAT/RUX (*n* = 33)
Age, median, years	67.0	70.0	69.0	70.0
Sex, *n* (%)				
Male	52 (78.8)	18 (46.2)	55 (76.4)	16 (48.5)
Female	14 (21.2)	21 (53.8)	17 (23.6)	17 (51.5)
DIPSS risk category, *n* (%)				
Intermediate-1	5 (7.6)	7 (17.9)	6 (8.3)	8 (24.2)
Intermediate-2	44 (66.7)	24 (61.5)	48 (66.7)	17 (51.5)
High	17 (25.8)	8 (20.5)	18 (25.0)	8 (24.2)
Total symptom score, mean (SD)	16.7 (11.2)	20.7 (15.7)	17.5 (11.7)	21.0 (16.3)
ECOG performance status, *n* (%)				
0	19 (28.8)	15 (38.5)	23 (31.9)	11 (33.3)
1	44 (66.7)	18 (46.2)	44 (61.1)	16 (48.5)
2	3 (4.5)	6 (15.4)	5 (6.9)	6 (18.2)
Duration of prior ruxolitinib treatment, mean (SD), weeks	62.7 (63.3)	62.5 (56.2)	64.6 (61.8)	59.5 (56.6)
*JAK2* V617F mutation, *n* (%)^a^				
Positive	43 (65.2)	27 (69.2)	47 (65.3)	23 (69.7)
Negative	22 (33.3)	9 (23.1)	23 (31.9)	7 (21.2)
TI, *n* (%)^b^	5 (7.6)	9 (23.1)	0	0
TD, *n* (%)^c^	52 (78.8)	25 (64.1)	58 (80.6)	27 (81.8)
TR, *n* (%)^d^	9 (13.6)	5 (12.8)	14 (19.4)	6 (18.2)
Hb level				
*n*	66	39	72	33
Mean (SD), g/L	82 (9)	88 (8)	86 (13)	87 (10)
< 100 g/L, *n* (%)	66 (100)	39 (100)	61 (84.7)	30 (90.9)
Platelet count				
*n*	64	39	70	33
Mean (SD), × 10^9^/L	186.4 (161.6)	123.5 (95.4)	190.8 (159.0)	119.4 (93.0)
Absolute neutrophil count				
*n*	66	38	72	33
Mean (SD), × 10^9^/L	8.8 (13.1)	7.3 (9.4)	10.3 (15.0)	6.0 (7.3)
EPO level				
*n*	63	37	68	32
Mean (SD), IU/L	442.6 (503.6)	281.3 (438.0)	410.4 (495.2)	350.7 (487.0)
< 100 IU/L, *n* (%)	19 (28.8)	19 (48.7)	22 (30.6)	13 (39.4)
< 500 IU/L, *n* (%)	45 (68.2)	31 (79.5)	50 (69.4)	25 (75.8)
Ruxolitinib and/or anemia supportive agents received, *n* (%)^e^				
Ruxolitinib only	N/A	18 (46.2)	N/A	15 (45.5)
Danazol only^f^		2 (5.1)		1 (3.0)
Ruxolitinib + danazol		1 (2.6)		1 (3.0)
Prednisolone only^f^		1 (2.6)		1 (3.0)
Ruxolitinib + prednisolone		1 (2.6)		1 (3.0)
ESA only		1 (2.6)		0
Ruxolitinib + ESA^g^		3 (7.7)		2 (6.0)
Lenalidomide only^h^		0		0
Ruxolitinib + lenalidomide^h^		0		0

*BAT/RUX* best available therapy/ruxolitinib, *DIPSS* Dynamic International Prognostic Scoring System, *ECOG* Eastern Cooperative Oncology Group, *EPO* erythropoietin, *ESA* erythropoiesis-stimulating agent, *Hb* hemoglobin, *JAK* Janus kinase, *N/A* not applicable, *RBC* red blood cell, *TD* transfusion dependent, *TI* transfusion independent, *TR* transfusion requiring

^a^In the Hb < 100 g/L subgroup, 65 of 66 patients in the momelotinib arm and 36 of 39 in the BAT/RUX arm were previously assessed for the *JAK2* V617F mutation. In the non-TI subgroup, 70 of 72 patients in the momelotinib arm and 30 of 33 in the BAT/RUX arm were previously assessed

^b^Defined as no RBC transfusions and no Hb of < 80 g/L in the previous 12 weeks

^c^Defined as ≥ 4 units of RBC transfusions or an Hb of < 80 g/L in the previous 8 weeks

^d^Defined as not meeting the criteria for transfusion independence or dependence

^e^Of the patients who received ruxolitinib in the BAT/RUX arm (34 in the Hb < 100 g/L subgroup, 29 in the non-TI subgroup), the remaining 8 and 9 patients, respectively, not represented here received ruxolitinib plus another therapy not directed at anemia supportive care

^f^One patient received both danazol and prednisolone

^g^A total of 5 patients received ESAs with or without ruxolitinib; 1 of these patients received ESAs plus ruxolitinib but did not have Hb of < 100 g/L or non-TI at baseline

^h^No patients received lenalidomide, but it is included for completeness

At baseline, 66 of 104 patients (63.5%) in the momelotinib arm and 39 of 52 patients (75.0%) in the BAT/ruxolitinib arm had Hb of < 100 g/L. In this subgroup, 86.7% had some transfusion need at baseline [i.e., were transfusion dependent (TD) or TR]; however, 14 patients were TI (5 in the momelotinib arm, 9 in the BAT/ruxolitinib arm) despite their moderate-to-severe anemia (Table 1).

In contrast, by definition, all patients in the baseline non-TI subgroup, which included 72 of 104 (69.2%) in the momelotinib arm and 33 of 52 (63.5%) in the BAT/ruxolitinib arm, had some transfusion need at baseline. In this subgroup, 81.0% met the criteria for transfusion dependence; the remaining 19.0% were TR. Despite their transfusion needs, 14 patients in this subgroup (11 in the momelotinib arm, 3 in the BAT/ruxolitinib arm) had baseline Hb of ≥ 100 g/L (Table 1).

In both subgroups, all patients in the momelotinib arm received a starting dose of 200 mg daily. In the BAT arm, 34 of 39 patients (87.2%) in the Hb < 100 g/L subgroup and 29 of 33 (87.9%) in the non-TI subgroup received ruxolitinib, alone or in combination with other BAT (Table 1); 59% of patients who received ruxolitinib in both subgroups [Hb < 100 g/L: 20 of 34 (58.8%); non-TI: 17 of 29 (58.6%)] received a baseline ruxolitinib dose of ≤ 10 mg twice daily. Near-full mean daily doses of momelotinib were maintained through week 24, while the percentage of patients receiving lower-dose ruxolitinib continued to increase over time in both subgroups (Supplementary Material). Overall safety results in these subgroups were consistent with those previously reported for the ITT population (Supplementary Material) [9], and no new or unexpected safety signals were observed.

Anemia supportive therapies, administered alone or in combination with ruxolitinib, in the BAT/ruxolitinib arm in these subgroups are shown in Table 1. In the overall BAT/ruxolitinib arm, the most common were ESAs, which were administered to 5 patients (4 of these patients also received ruxolitinib). At baseline, 3 of these ESA-treated patients were TI (Hb: 98, 103, and 96 g/L), 1 was TR (Hb: 85 g/L), and 1 was TD (Hb: 76 g/L).

### Anemia-Related Efficacy

**Fig. 1:**
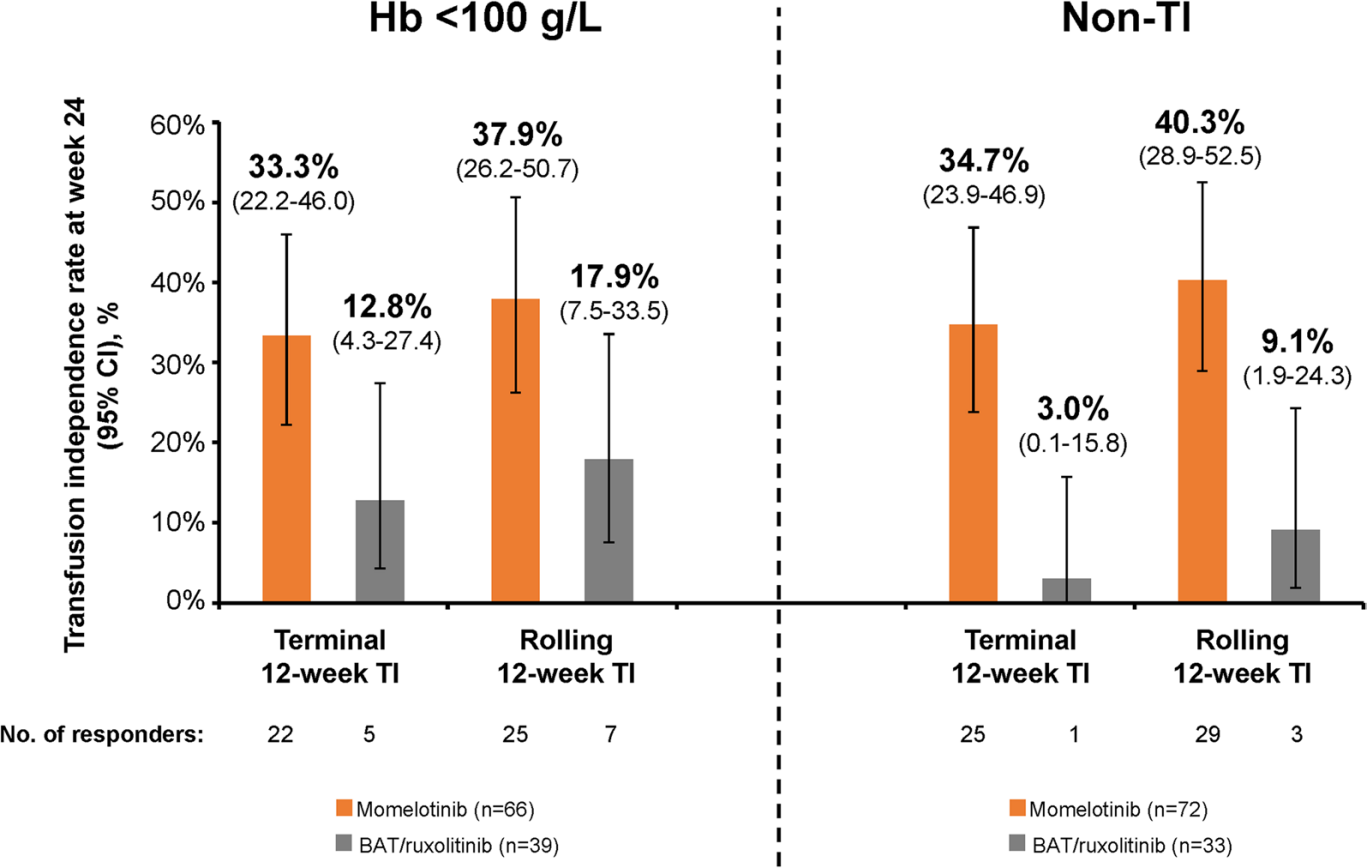
Week 24 transfusion independence in the baseline Hb < 100 g/L and non-TI subgroups. Transfusion independence at week 24 (terminal 12-week definition; defined as no RBC transfusions and no Hb of < 80 g/L in the last 12 weeks before week 24) or by week 24 (rolling 12-week definition; defined as no RBC transfusions and no Hb of < 80 g/L during any 12-week period through week 24). *BAT* best available therapy, *Hb* hemoglobin, *RBC* red blood cell, *TI* transfusion independent

In both subgroups of interest, week 24 transfusion independence rates were higher with momelotinib versus BAT/ruxolitinib. Among patients with baseline Hb of < 100 g/L, 22 (33.3%) were TI at week 24 with momelotinib per the prespecified terminal 12-week definition; 25 (37.9%) were TI by week 24 per the rolling 12-week definition (Fig. 1). In contrast, only 5 (12.8%) and 7 (17.9%) in this subgroup were TI per the week 24 terminal and rolling definitions, respectively, with BAT/ruxolitinib.

Similarly, 25 patients (34.7%) in the non-TI subgroup were TI with momelotinib per the terminal 12-week definition and 29 (40.3%) were TI per the rolling 12-week definition versus only 1 (3.0%) and 3 (9.1%) with BAT/ruxolitinib, respectively (Fig. 1). Transfusion independence rates at week 24 based on baseline erythropoietin (EPO) level were also assessed within these subgroups of interest; there was no influence of baseline EPO levels on transfusion independence rates with momelotinib, and response rates were consistently higher with momelotinib versus BAT/ruxolitinib (Supplementary Material).

**Fig. 2:**
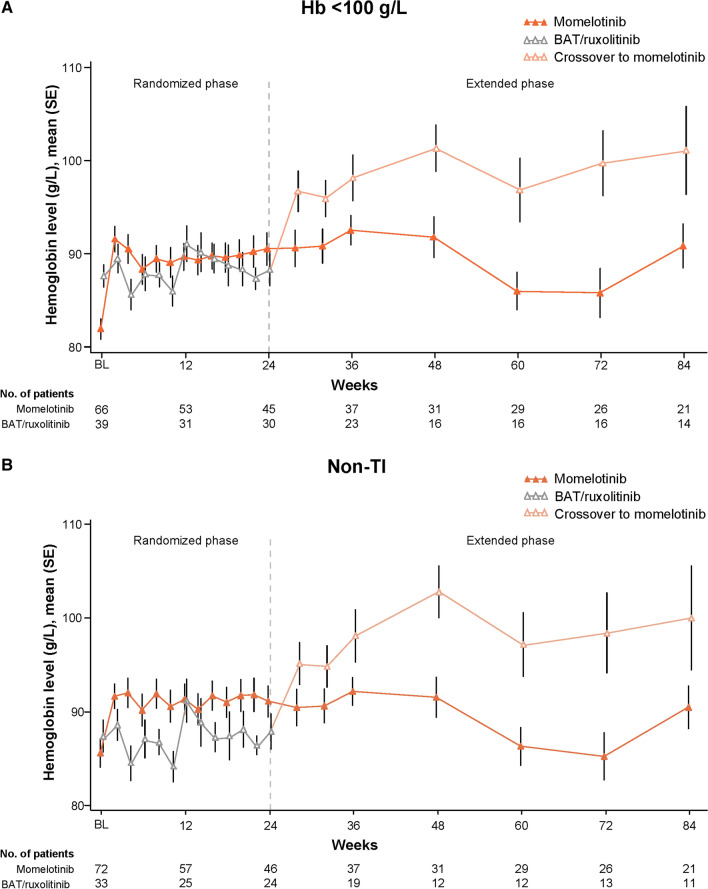
Mean Hb levels over time in the baseline Hb < 100 g/L (**A**) and non-TI (**B**) subgroups. Results through week 84 are shown for illustrative purposes, although the study continued beyond this time point. *BAT* best available therapy, *BL* baseline, *Hb* hemoglobin, *TI* transfusion independent

Similar to the overall ITT population [9], mean Hb levels in these subgroups improved rapidly with momelotinib and remained higher over time than in those treated with BAT/ruxolitinib, including additional improvement in the BAT/ruxolitinib groups after crossover to momelotinib at week 24 (Fig. 2). These Hb improvements in the momelotinib versus BAT/ruxolitinib groups were apparent despite median transfusion rates through week 24 being comparable or lower with momelotinib [units/month (range), Hb < 100 g/L: 1.6 (0–8.2) vs. 1.4 (0–7.6); non-TI: 1.2 (0–8.7) vs. 1.8 (0–7.6)].

### Spleen, Symptom, and Dual/Triple Responses

**Fig. 3:**
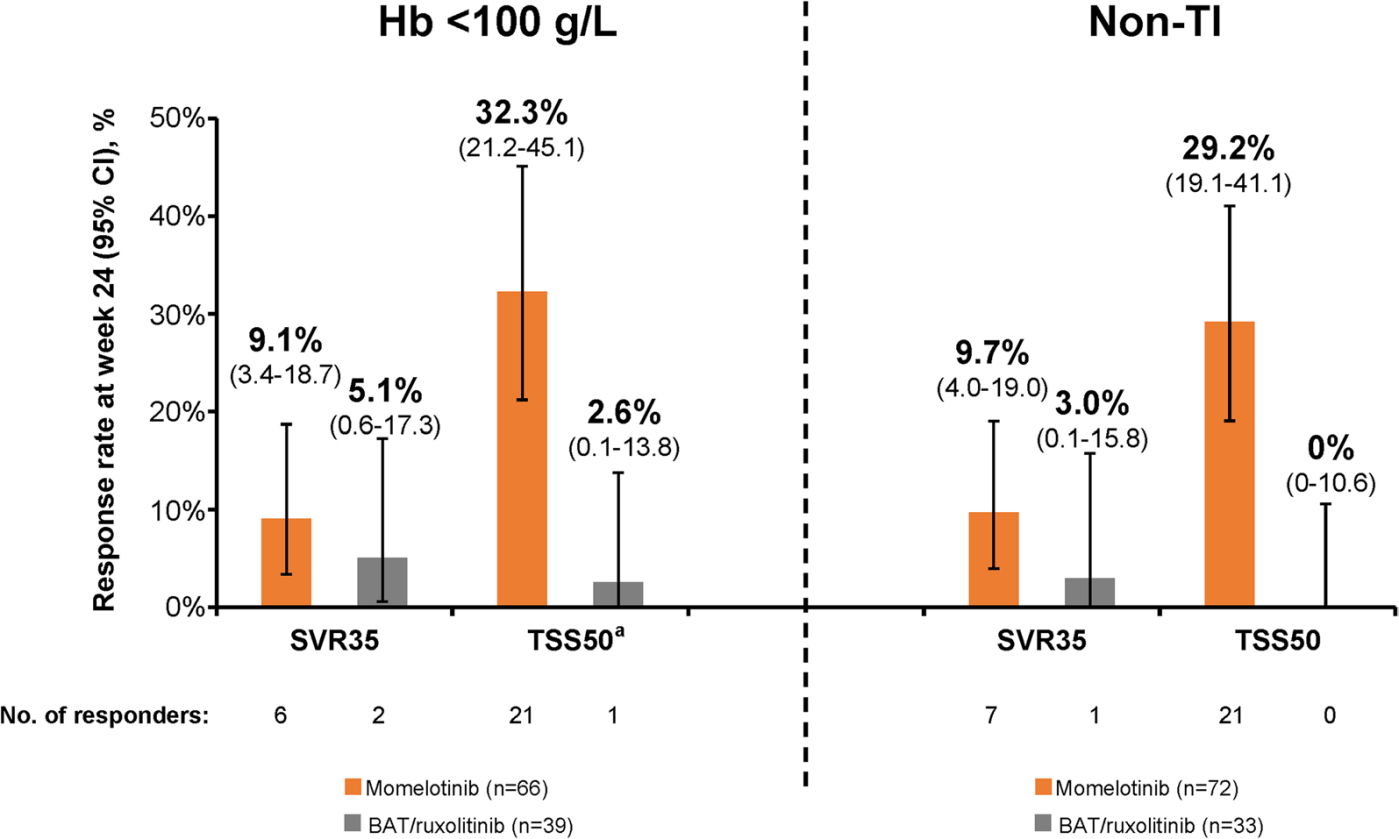
SVR35 and TSS50 at week 24 in the baseline Hb < 100 g/L and non-TI subgroups. For TSS50, response rates are based on the number of patients evaluable for TSS at week 24. *BAT* best available therapy, *Hb* hemoglobin, *SVR35* spleen volume reduction ≥ 35%, *TI* transfusion independent, *TSS50* total symptom score reduction ≥ 50%. ^a^In the Hb < 100 g/L subgroup, *n* = 65 for the momelotinib arm and *n* = 38 for the BAT/ruxolitinib arm

Although these subgroups of interest were selected primarily to evaluate the anemia-related benefits of momelotinib versus BAT/ruxolitinib, rates of splenic response [Hb < 100 g/L: 6 (9.1%) vs. 2 (5.1%); non-TI: 7 (9.7%) vs. 1 (3.0%)] and symptom response [Hb < 100 g/L: 21 (32.3%) vs. 1 (2.6%); non-TI: 21 (29.2%) vs. 0] at week 24 were also higher with momelotinib (Fig. 3).

Many responders with momelotinib achieved 2 or all 3 endpoints (splenic, symptom, and transfusion independence response), including 15 of 33 responders (45.5%) in the Hb < 100 g/L subgroup and 16 of 36 responders (44.4%) in the non-TI subgroup; there were no dual or triple responses in the BAT/ruxolitinib arm in either subgroup. In the Hb < 100 g/L subgroup, 3 of 6 splenic responders (50.0%) and 10 of 21 symptom responders (47.6%) with momelotinib were also TI at week 24. Similarly, in the non-TI subgroup, 4 of 7 splenic responders (57.1%) and 10 of 21 symptom responders (47.6%) with momelotinib were also TI at week 24 (Supplementary Material).

### Efficacy in Patients Who Received ESAs

Among the 5 patients in the overall BAT/ruxolitinib arm who received ESAs with or without ruxolitinib, there were 3 single responses (1 for each endpoint: splenic, symptom, and transfusion independence response). The ESA-treated patient who achieved a symptom response at week 24 did not receive ruxolitinib, while the splenic and transfusion independence responders received an ESA in combination with ruxolitinib. Both the splenic and symptom responders were included in the Hb < 100 g/L subgroup, while the transfusion independence responder was not in either subgroup of interest. Thus, no patients with Hb of < 100 g/L or who were non-TI at baseline achieved transfusion independence at week 24 with ESAs. Furthermore, no patients in these subgroups of interest who received other anemia supportive therapies achieved transfusion independence at week 24.

## Discussion

These exploratory analyses of the phase 3 SIMPLIFY-2 trial focused on JAK inhibitor-experienced patient subgroups for whom anemia management is typically a key consideration: patients with Hb of < 100 g/L and patients who were non-TI. In both populations, anemia-related benefits and continued spleen and symptom control were greater with momelotinib than with BAT/ruxolitinib, suggesting that it may be advantageous to switch to momelotinib versus continuing ruxolitinib and managing anemia with supportive care. Because there was no washout of prior ruxolitinib in SIMPLIFY-2, these results complement a recent analysis of the phase 3 SIMPLIFY-1 trial illustrating that patients can be immediately transitioned from ruxolitinib to momelotinib at full dose with no safety concerns, derive rapid anemia benefits, and maintain splenic and symptom control [12].

The subgroups in these analyses comprise similar but not completely overlapping patient populations. Hb of < 100 g/L broadly constitutes moderate-to-severe anemia, and while supportive therapies are typically considered at this threshold, the introduction of RBC transfusions may be reserved for more severely anemic individuals [3, 13–17]. Thus, it is possible to encounter patients with Hb of < 100 g/L who require anemia-directed therapy but are nevertheless TI (14 patients in the present analysis). Conversely, as transfusions may themselves increase Hb levels, there may be patients with Hb of ≥ 100 g/L who are nevertheless TD or TR (as is the case for a separate group of 14 patients in the present analysis). While anemia-related benefits are of particular importance in the management of both of these subgroups, the goals of treatment may vary (e.g., Hb improvement versus transfusion burden reduction). Momelotinib was associated with comprehensive anemia benefits, including higher mean Hb levels over time and increased rates of week 24 transfusion independence, versus BAT/ruxolitinib in both subgroups.

Anemia supportive therapies do not address the underlying mechanism of anemia in myelofibrosis and are unable to halt its progression; however, adding these supportive therapies to ongoing treatment with a JAK inhibitor such as ruxolitinib may be perceived as a preferred approach, particularly in patients who are otherwise experiencing symptom and spleen control [1, 3, 18]. In fact, our analyses suggest that this approach provides not only limited anemia benefit but also lower symptom and spleen control compared with switching to momelotinib. Consistent with the ITT analysis, in which the primary superiority endpoint was not met, splenic response rates were low across treatment arms and subgroups, likely attributable to the lack of washout from prior ruxolitinib [9]. However, splenic response rates with momelotinib in these subgroups were higher than in the ITT population (ITT, 6.7%; subgroups, 9.1% and 9.7%), while those with BAT/ruxolitinib were lower (ITT, 5.8%; subgroups, 5.1% and 3.0%). A similar trend was observed with symptom responses; notably, no patients in the non-TI subgroup, and only 1 in the Hb < 100 g/L subgroup, treated with BAT/ruxolitinib had a symptom response, while approximately 30% did with momelotinib. These poor splenic and symptom response rates in the BAT/ruxolitinib arm may be the result of suboptimal ruxolitinib dosing, as most patients in these subgroups received ≤ 20 mg daily. These observations are consistent with recent real-world analyses of ruxolitinib, which found lower overall doses, splenic and symptom response rates, and survival in patients with cytopenias, primarily anemia [19]. In contrast, most patients in the present analysis received full daily doses of momelotinib, which not only provides spleen and symptom benefits through inhibition of JAK1/JAK2 but directly addresses anemia in myelofibrosis through inhibition of ACVR1, leading to increased serum iron availability and erythropoiesis [3, 20]. Thus, the decision to continue first-line treatment or switch may be informed in part by the dose of the initial JAK inhibitor that can be maintained and the corresponding depth and durability of spleen and symptom responses.

The prespecified terminal 12-week definition of transfusion independence in SIMPLIFY-2 [9] ensures the durability of the rate reported at week 24, as it requires patients to have remained transfusion-free for at least the previous 12 weeks. In contrast, the rolling 12-week definition [21] is less stringent, as patients may achieve transfusion independence over any 12-week period through week 24; even if this independence is later lost, they will still be counted as responders at week 24. The fact that week 24 transfusion independence rates with momelotinib were similar by the terminal and rolling 12-week definitions (e.g., 40.3% vs. 34.7% in the non-TI subgroup) provides further evidence of durability, as it indicates that few patients lost response with momelotinib once achieved; in contrast, few patients met the strict terminal 12-week criteria for transfusion independence in the BAT/ruxolitinib arm.

The most common anemia supportive therapy, other than transfusions, in the BAT/ruxolitinib arm of SIMPLIFY-2 was ESAs (5 patients). Current guidelines recommend ESAs primarily for patients with serum EPO levels of < 500 IU/L [11], with responses more common in those with levels of < 125 IU/L [16]. In the subgroups evaluated in the present analysis, more patients in the BAT/ruxolitinib arm versus the momelotinib arm had baseline EPO levels of < 500 IU/L, including approximately 40–50% versus 30% with levels of < 100 IU/L. However, transfusion independence responses with momelotinib were observed regardless of baseline EPO level, suggesting that this criterion should not impact consideration of momelotinib in patients who require an anemia benefit. Notably, no patients in either SIMPLIFY-2 subgroup of interest achieved transfusion independence at week 24 with ESAs.

The primary limitation of the present analyses is their post hoc and descriptive nature; SIMPLIFY-2 was not powered to prospectively evaluate momelotinib versus BAT/ruxolitinib in these patient subgroups; thus, statistical significance could not be determined. The crossover design of SIMPLIFY-2 also represents a limitation, as this precludes comparative analyses of endpoints such as duration of transfusion independence beyond week 24.

## Conclusion

Collectively, these data suggest that, in patients with moderate-to-severe anemia and/or in need of RBC transfusions, outcomes—notably anemia benefits, including week 24 transfusion independence rates, onset and duration of transfusion independence, median transfusion rates through week 24, and mean Hb levels over time—are improved by switching to momelotinib rather than continuing ruxolitinib and using ESAs or other supportive therapies to manage anemia.

### Supplementary Information

Below is the link to the electronic supplementary material.

Supplementary file1 (PDF 564 KB)

## Data Availability

The datasets generated during and/or analyzed during the current study are available from the corresponding author on reasonable request. Information on GSK’s data sharing commitments and requesting access to anonymized individual participant data and associated study documents can be found at https://www.gsk-studyregister.com/en/.
