# Supplementary material for: Momelotinib versus Continued Ruxolitinib or Best Available Therapy in JAK Inhibitor-Experienced Patients with Myelofibrosis and Anemia: Subgroup Analysis of SIMPLIFY-2
Source: Adv Ther. 2024 Jul 11;41(9):3722–35. doi: 10.1007/s12325-024-02928-4 (PMC11349857; doi:10.1007/s12325-024-02928-4)

Supplementary Material for:

Momelotinib vs continued ruxolitinib or best available therapy in JAK inhibitor–experienced patients with myelofibrosis and anemia: subgroup analysis of SIMPLIFY-2

Claire N. Harrison,[1] Alessandro M. Vannucchi,[2] Christian Recher,[3] Francesco Passamonti,[4] Aaron T. Gerds,[5] Juan Carlos Hernandez-Boluda,[6] Abdulraheem Yacoub,[7] Shireen Sirhan,[8] Catherine Ellis,[9] Bharat Patel,[9] Bryan Strouse,[9] Uwe Platzbecker[10]

[1]Guy's and St Thomas' NHS Foundation Trust, London, UK; [2]University of Florence, Florence, Italy; [3]University Hospital Center (CHU) of Toulouse, Toulouse, France; [4]University of Milan Statale, Milan, Italy; [5]Cleveland Clinic Taussig Cancer Institute, Cleveland, OH, USA; [6]Hospital Clìnico de Valencia-INCLIVA, University of Medicine, Valencia, Spain; [7]University of Kansas, Kansas City, KS, USA; [8]Jewish General Hospital, McGill University, Montreal, QC, Canada; [9]GSK plc, Philadelphia, PA, USA; [10]University Hospital Leipzig, Leipzig, Germany

Corresponding Author: Claire Harrison, claire.harrison@gstt.nhs.uk

List of SIMPLIFY-2 Investigators

Canada: Vikas Gupta, Brian Leber, Elena Liew, Shireen Sirhan; **France:** Fiorenza Barraco, Nathalie Cambier, Viviane Dubruille, Jean-Jacques Kiladjian, Christian Recher, Jerome Rey, Vincent Ribrag, Philippe Rodon; **Germany:** Philipp Jost, Uwe Platzbecker, Andreas Reiter, Philippe Schafhausen, Christof Scheid, Cornelius Waller; **Israel:** Dina Ben Yehuda, Andrei Braester, Martin Ellis, Ilya Kirgner, Gilles Lugassy, Elena Mishchenko, Arnon Nagler, Meir Preis, Jacon Rowe; **Italy:** Giuliana Alimena, Michele Cavo, Agostino Cortelezzi, Gianluca Gaidano, Marco Gobi, Francesco Passamonti, Marco Ruggeri, Alessandro Vannucchi; **Spain:** Rose Ayala, Francisco Cervantes, Pilar Giraldo, Juan Carlos Hernandez-Boluda, Jesus Maria Hernandez Rivas, Cristina Motillo, Blanca Xicoy; **UK:** David Bowen, Joanne Ewing, Mamta Garg, Claire Harrison, Dragana Milojkovic, Timothy Somervaille; **USA:** Murat Arcasoy, Maria Baer, Dmitriy Berenzon, Randall Brown, Catherine Cargo, Salman Fazal, Aaron Gerds, Mark Heaney, Daniel Huang, Carole Miller, Stephen Oh, Gary Schiller, Elliot Winton, Srdan Verstovsek, Abdulraheem Yacoub, Cecilia Yi

List of Institutional Review Boards/Ethics Committees

Study Site Number	Institutional Review Board/Ethics Committee Name
9012	Institutional Helsinki Committee, Shaare Zedek Medical Center
9865	Carmel Medical Center Helsinki Committee/IRB Carmel MC Helsinki Committee
9804	Institutional Helsinki Committee, The Chaim Sheba Medical Center
15064	Institutional Helsinki Committee
8483	Institutional Helsinki Committee, Barzilai Medical Center
8229	Institutional Helsinki Committee, Tel-Aviv Sourasky Medical Center
9806	Institutional Helsinki Committee, Rabin Medical Center, -Belinson Campus
9805	Institutional Helsinki Committee, Meir Medical Center
8766	Institutional Helsinki Committee,Galilee Medical Center
8195	Institutional Helsinki Committee, Hadassah Medical Center

8861<br>9008<br>8974<br>9059<br>8768<br>9024<br>8680<br>8689<br>8828<br>8872<br>3891	CPP ILE DE France 1
8834<br>8982<br>8781<br>8886<br>8981<br>9579<br>9365	Ethik-Kommission der Ärztekammer Westfalen- Lippe und der Westfälischen Wilhelms-Universität Münster
15480<br>8781	Ethik-Kommission an der Technischen Universität Dresden
8834	Ethik-Kommission der Ärztekammer Westfalen- Lippe und der Medizinischen Fakultät der WWU Münster
8886	Universitätsklinikum Mannheim gGmbH, Medizinische Ethik-Kommission II
8981	Ethik-Kommission der Ärztekammer Hamburg
8982	Ethik-Kommission der Fakultät für Medizin der Technischen Universität München
9365	Ethikkommission der Albert-Ludwigs-Universität Freiburg
9579	Ethikkommission der Medizinischen Fakultät der Universität zu Köln
9400<br>7706<br>8747<br>10344<br>7875<br>5427<br>8780<br>8783	Comitato Etico Area Vasta Azienda Ospedaliero-Universitaria Careggi Pad. 3 - Nuovo Ingresso Careggi (NIC) – Didattica

8685	
9400	Comitato Etico dell'Università "Sapienza" (Policlinico Universitario Umberto I - A.O. Sant' Andrea)
7706	Comitato Etico di Area Vasta Emilia Centro Azienda Ospedaliero-Universitaria Policlinico Sant'Orsola – Malpighi di Bologna
8747	Comitato Etico Interaziendale A.O.U. San Luigi Gonzaga
10344	Comitato Etico Milano Area 2, Fondazione IRCCS Ca' Granda Ospedale Maggiore Policlinico
7875	Comitato Etico Interaziendale AOU Maggiore della Carita di Novara
5427	Comitato Etico Regionale della Liguria (Sezione 2), Ospedale Policlinico San Martino
8780	President of Comitato Etico dell'Insubria
8783	Comitato Etico per la Sperimentazione Clinica della Provincia di Vicenza
9366<br>8950<br>8958<br>8771<br>9876<br>9914	CEIC Hospital Clinic de Barcelona-CEC
10015<br>9336<br>9013<br>9335<br>9102<br>9361	West Midlands – Edgbaston Research Ethics Committee (REC)
8156	Sir Mortimer B.Davis-Jewish General Hospital
9920	University of Alberta Health Research Ethics Board
8949	Hamilton Integrated Research Ethics Board
8155	University Health Network Research Ethics Board

9282	University of Kansas Human Subjects Committee
9293	Brookdale University Hospital and Medical Center
5576	The University of Texas MD Anderson Cancer Center, Institution Review Board
9334	Rush University Medical Center IRB
8863	UCLA Office of Human Research Protection Program
7034	Washington University HRPO
8808	Columbia University Medical Center IRB
9887	The Cleveland Clinic Institutional Review Board Cleveland Clinic Foundation
10948	Cleveland Clinic Foundation, Institutional Review Board
9748<br>8874<br>8956<br>8810	Quorum Review, Inc.
8881<br>9704	Western Institutional Review Board (WIRB)

Table S1. Safety summary and most common AEs during the randomized period in the baseline Hb <100 g/L and non-TI subgroups

n (%)	Baseline Hb <100 g/L		Baseline non-TI	
	**Momelotinib (n=66)**	**BAT/RUX (n=39)**	**Momelotinib (n=72)**	**BAT/RUX (n=33)**
**Any-grade TEAE**	66 (100)	35 (89.7)	71 (98.6)	30 (90.9)
Grade ≥3 TEAE	40 (60.6)	18 (46.2)	45 (62.5)	14 (42.4)
**Treatment-related AE**	49 (74.2)	15 (38.5)	52 (72.2)	11 (33.3)
Grade ≥3 treatment-related AE	20 (30.3)	8 (20.5)	22 (30.6)	6 (18.2)
**Serious TEAE**	23 (34.8)	9 (23.1)	27 (37.5)	8 (24.2)
Treatment-related serious AE	7 (10.6)	2 (5.1)	9 (12.5)	1 (3.0)
**TEAE leading to discontinuation[a]**	14 (21.2)	1 (2.6)	17 (23.6)	1 (3.0)
**TEAE leading to dose reduction/interruption**	9 (13.6)	8 (20.5)	9 (12.5)	6 (18.2)
**TEAE leading to death**	4 (6.1)	4 (10.3)	5 (6.9)	3 (9.1)
**Most common TEAEs (>10% of the momelotinib arm in either subgroup)**				
Diarrhea	21 (31.8)	5 (12.8)	23 (31.9)	5 (15.2)
Anemia	13 (19.7)	10 (25.6)	13 (18.1)	5 (15.2)
Asthenia	13 (19.7)	8 (20.5)	15 (20.8)	7 (21.2)
Nausea	12 (18.2)	3 (7.7)	12 (16.7)	1 (3.0)
Dizziness	11 (16.7)	3 (7.7)	11 (15.3)	2 (6.1)
Pyrexia	11 (16.7)	3 (7.7)	14 (19.4)	4 (12.1)
Thrombocytopenia	11 (16.7)	4 (10.3)	11 (15.3)	4 (12.1)
Abdominal pain	10 (15.2)	5 (12.8)	11 (15.3)	3 (9.1)
Fatigue	10 (15.2)	7 (17.9)	9 (12.5)	6 (18.2)
Pruritus	10 (15.2)	3 (7.7)	10 (13.9)	2 (6.1)
Cough	9 (13.6)	4 (10.3)	11 (15.3)	3 (9.1)
Arthralgia	8 (12.1)	3 (7.7)	8 (11.1)	2 (6.1)
Dyspnea	8 (12.1)	6 (15.4)	10 (13.9)	4 (12.1)
Headache	8 (12.1)	3 (7.7)	10 (13.9)	1 (3.0)
Peripheral edema	8 (12.1)	5 (12.8)	10 (13.9)	4 (12.1)
Dyspepsia	7 (10.6)	0	8 (11.1)	0
Neutropenia	7 (10.6)	1 (2.6)	7 (9.7)	0
Weight decreased	7 (10.6)	1 (2.6)	8 (11.1)	2 (6.1)
Upper respiratory tract infection	7 (10.6)	3 (7.7)	5 (6.9)	2 (6.1)
Vitamin B_1 deficiency[b]	7 (10.6)	1 (2.6)	6 (8.3)	1 (3.0)
Peripheral sensory neuropathy	6 (9.1)	0	8 (11.1)	0
Urinary tract infection	6 (9.1)	3 (7.7)	9 (12.5)	4 (12.1)

AE, adverse event; BAT/RUX, best available therapy/ruxolitinib; Hb, hemoglobin; TEAE, treatment-emergent adverse event; TI, transfusion independent.

[a] Discontinuation of BAT was inconsistently reported because changes in therapy or intentional absence of therapy were permissible options for this treatment group. [b] All AEs of decreased vitamin B_1/vitamin B_1 deficiency were grade 1/2; no AEs of Wernicke encephalopathy were reported.

Figure S1. Mean daily dose intensity over time in the baseline Hb <100 g/L and non-TI subgroups. Shown are mean daily doses of momelotinib (A and C) and ruxolitinib (in the BAT arm; B and D). BAT, best available therapy; Hb, hemoglobin; TI, transfusion independent.

A.

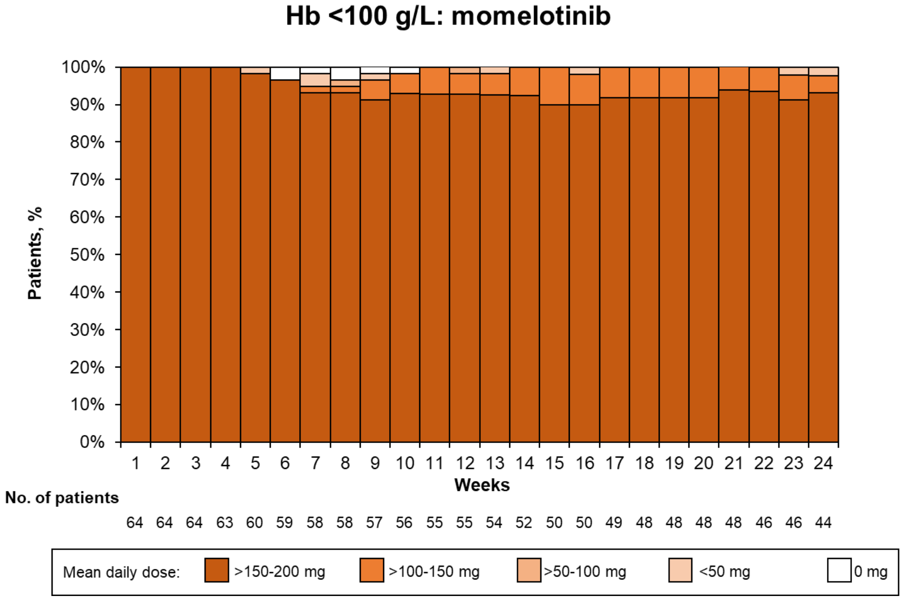

B.

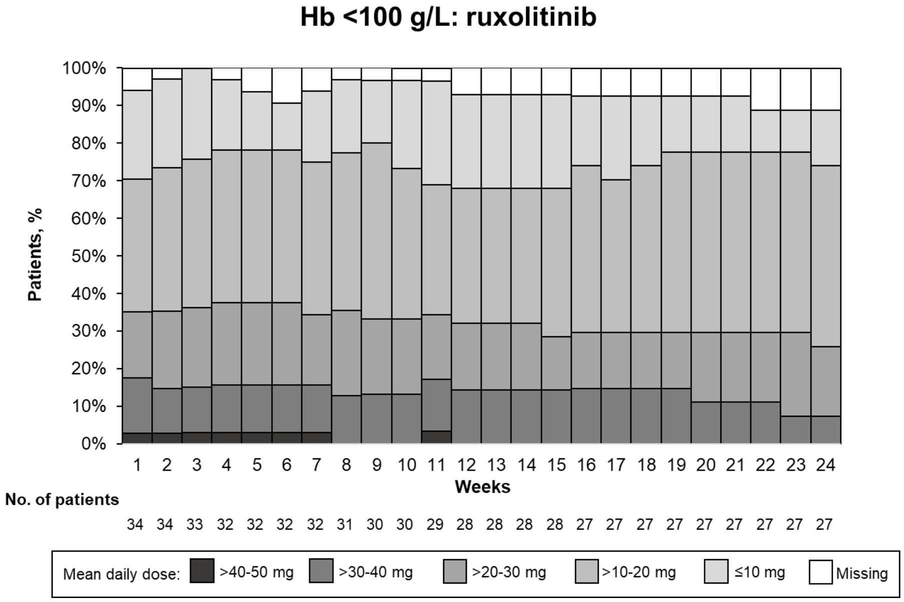

C.

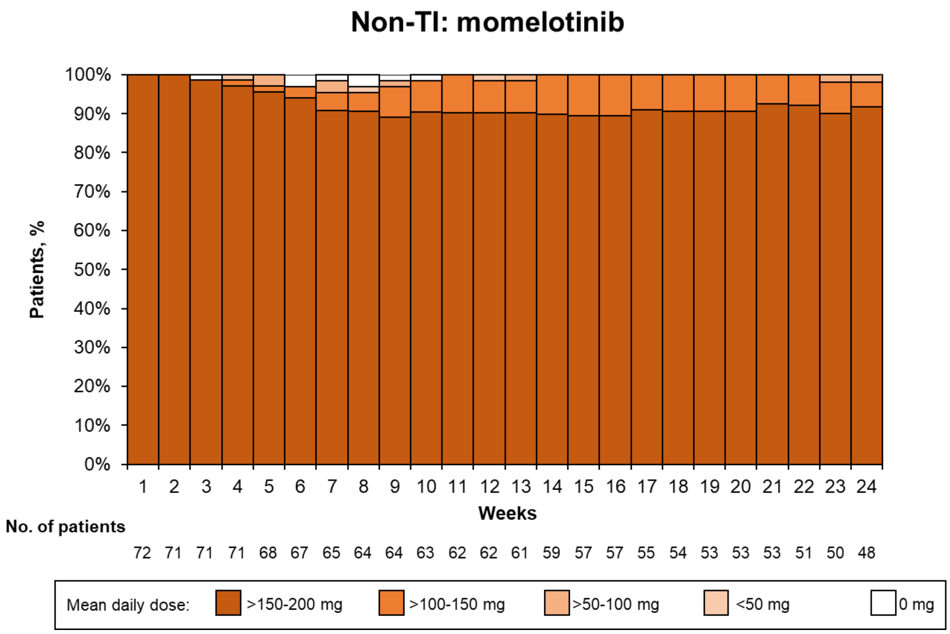
Non-TI: momelotinib
Patients, %
100%
90%
80%
70%
60%
50%
40%
30%
20%
10%
0%
1 2 3 4 5 6 7 8 9 10 11 12 13 14 15 16 17 18 19 20 21 22 23 24
Weeks
No. of patients
72 71 71 71 68 67 65 64 64 63 62 62 61 59 57 57 55 54 53 53 53 51 50 48
Mean daily dose:
>150-200 mg
>100-150 mg
>50-100 mg
<50 mg
0 mg

D.

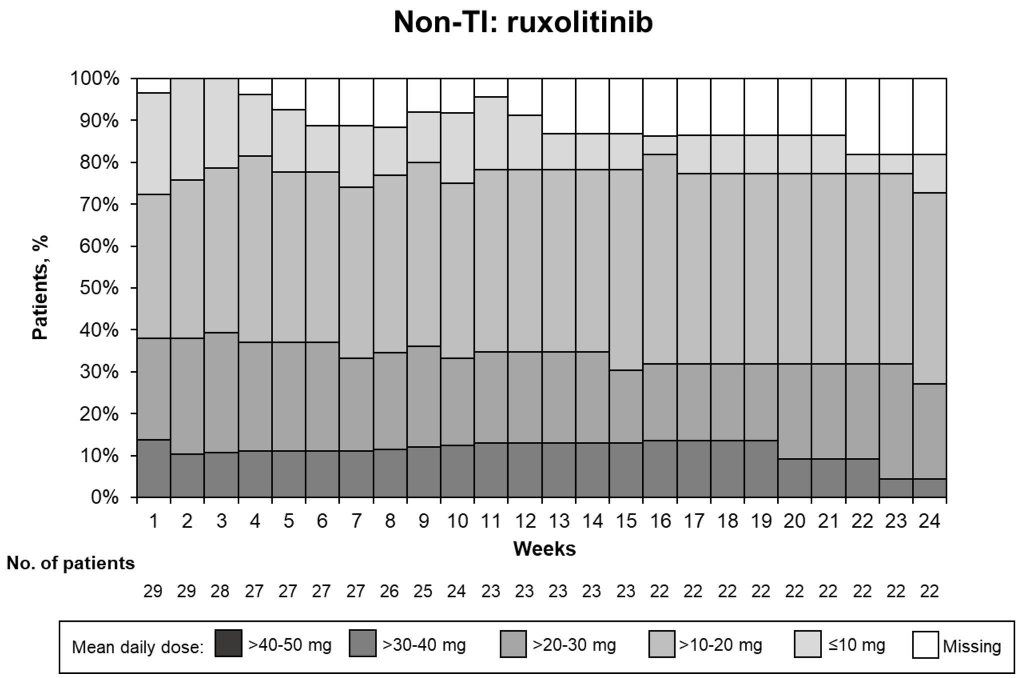
Non-TI: ruxolitinib
Patients, %
100%
90%
80%
70%
60%
50%
40%
30%
20%
10%
0%
1 2 3 4 5 6 7 8 9 10 11 12 13 14 15 16 17 18 19 20 21 22 23 24
Weeks
No. of patients
29 29 28 27 27 27 27 26 25 24 23 23 23 23 23 22 22 22 22 22 22 22 22 22
Mean daily dose:
>40-50 mg
>30-40 mg
>20-30 mg
>10-20 mg
≤10 mg
Missing

Figure S2. Week 24 transfusion independence by baseline EPO level (< or ≥100, < or ≥500 IU/L) in the baseline Hb <100 g/L (A) and non-TI (B) subgroups.
Transfusion independence at week 24 (terminal 12-week definition; defined as no RBC transfusions and no Hb of <80 g/L in the last 12 weeks before week 24) or by week 24 (rolling 12-week definition; defined as no RBC transfusions and no Hb of <80 g/L during any 12-week period through week 24). BAT, best available therapy; EPO, erythropoietin; Hb, hemoglobin; RBC, red blood cell; TI, transfusion independent.

A.

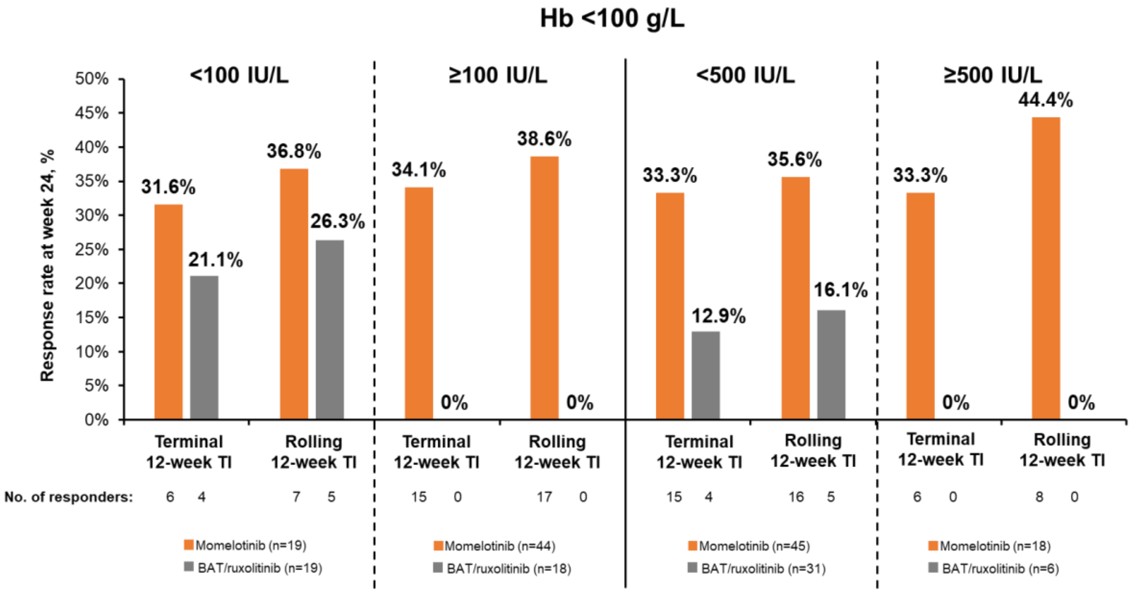

B.

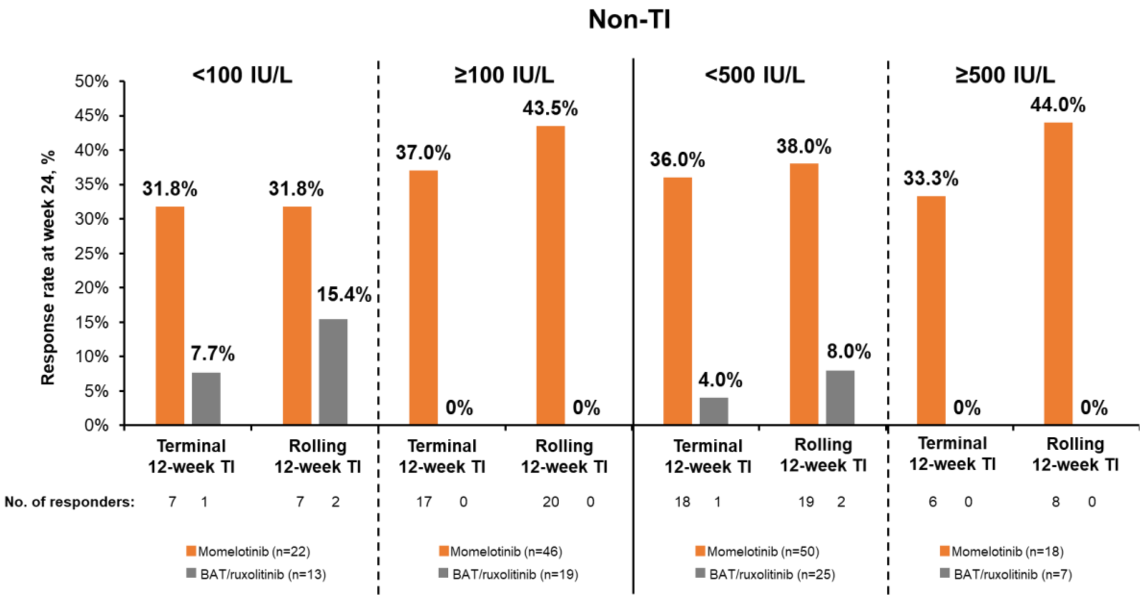

Figure S3. Dual and triple response rates (SVR35, TSS50, and TI-R [terminal 12-week definition]) in the baseline Hb <100 g/L (A) and non-TI (B) subgroups. BAT, best available therapy; Hb, hemoglobin; SVR35, spleen volume reduction ≥35%; TI, transfusion independent; TI-R, transfusion independence response; TSS50, Total Symptom Score reduction ≥50%.

A.

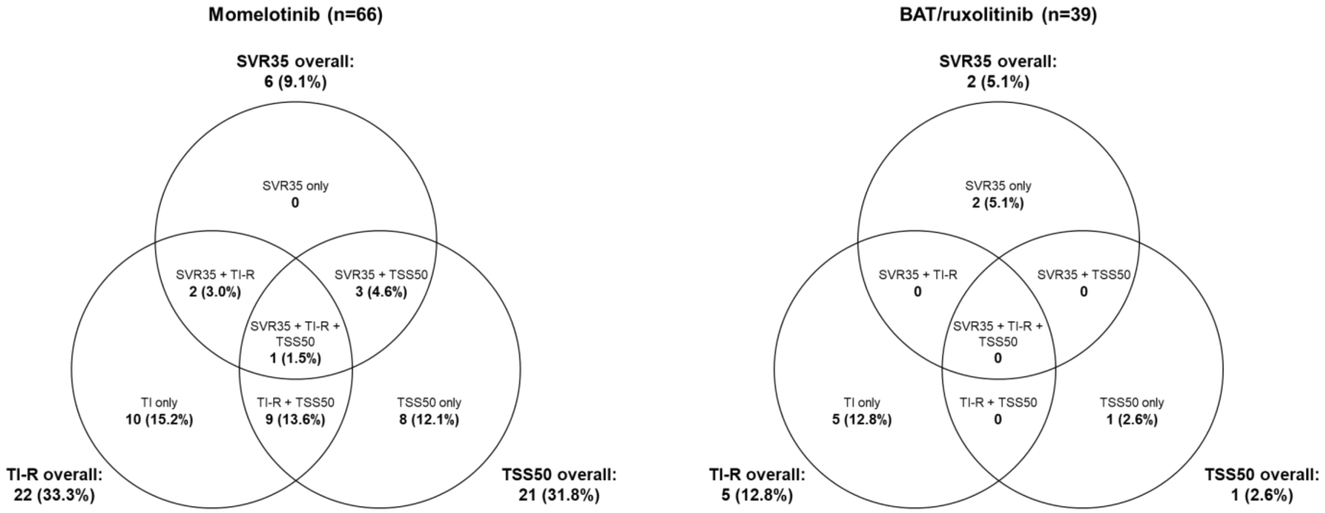

B.

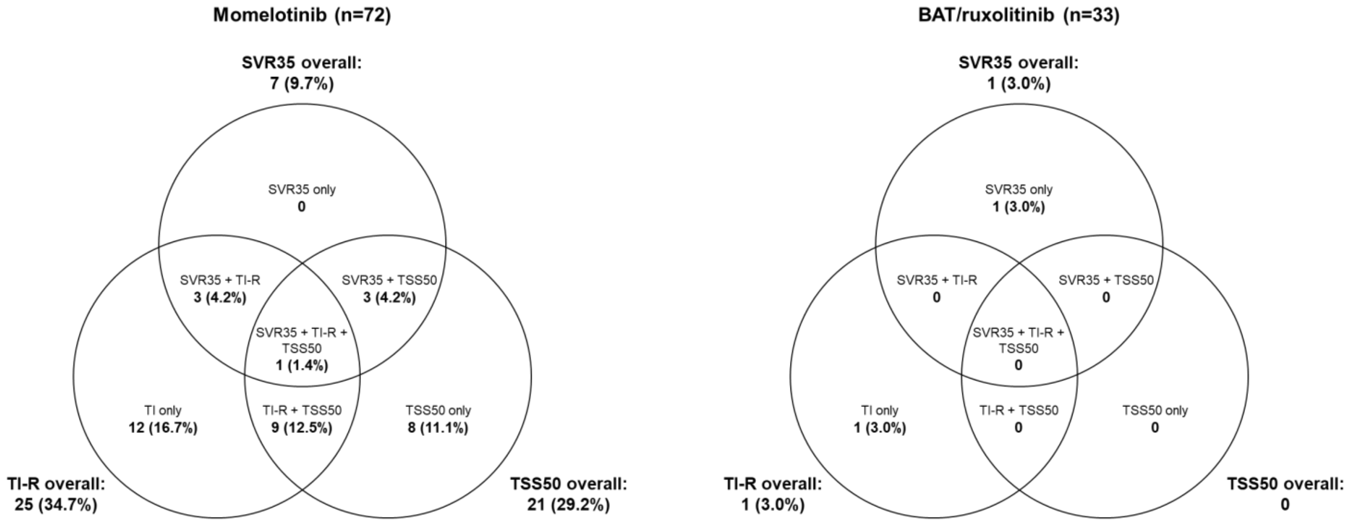

Supplement: Supplementary file 1 — Supplementary file1 (PDF 564 KB) [file 12325_2024_2928_MOESM1_ESM.pdf]
